# Disruption of Extracellular Matrix and Perineuronal Nets Modulates Extracellular Space Volume and Geometry

**DOI:** 10.1523/JNEUROSCI.0517-24.2024

**Published:** 2025-01-03

**Authors:** Eva Syková, Ivan Voříšek, Zenon Starčuk, Jiří Kratochvíla, Iveta Pavlova, Yuki Ichikawa, Jessica C. F. Kwok, Eva Kmoníčková, Svitlana Myronchenko, Tomáš Hromádka, Tomáš Smolek, Martin Avila, Neha Basheer, Norbert Žilka

**Affiliations:** ^1^Institute of Neuroimmunology, Slovak Academy of Science, Bratislava 84510, Slovakia; ^2^Institute of Scientific Instruments, Czech Academy of Sciences, Brno 61200, Czech Republic; ^3^School of Biomedical Sciences, Faculty of Biological Sciences, University of Leeds, Leeds LS2 9JT, United Kingdom; ^4^Department of Pharmacology, Second Faculty of Medicine, Charles University, Prague 15000, Czech Republic; ^5^Institute of Histology and Embryology of Mendoza, National University of Cuyo, National Scientific and Technical Research Council, Mendoza 5500, Argentina

**Keywords:** extracellular diffusion, extracellular matrix, extracellular transmission, hyaluronan synthase, perineuronal nets, plasticity

## Abstract

Extracellular matrix (ECM) is a network of macromolecules which has two forms—perineuronal nets (PNNs) and a diffuse ECM (dECM)—both influence brain development, synapse formation, neuroplasticity, CNS injury and progression of neurodegenerative diseases. ECM remodeling can influence extrasynaptic transmission, mediated by diffusion of neuroactive substances in the extracellular space (ECS). In this study we analyzed how disrupted PNNs and dECM influence brain diffusibility. Two months after oral treatment of rats with 4-methylumbelliferone (4-MU), an inhibitor of hyaluronan (HA) synthesis, we found downregulated staining for PNNs, HA, chondroitin sulfate proteoglycans, and glial fibrillary acidic protein. These changes were enhanced after 4 and 6 months and were reversible after a normal diet. Morphometric analysis further indicated atrophy of astrocytes. Using real-time iontophoretic method dysregulation of ECM resulted in increased ECS volume fraction *α* in the somatosensory cortex by 35%, from *α* = 0.20 in control rats to *α* = 0.27 after the 4-MU diet. Diffusion-weighted magnetic resonance imaging revealed a decrease of mean diffusivity and fractional anisotropy (FA) in the cortex, hippocampus, thalamus, pallidum, and spinal cord. This study shows the increase in ECS volume, a loss of FA, and changes in astrocytes due to modulation of PNNs and dECM that could affect extrasynaptic transmission, cell-to-cell communication, and neural plasticity.

## Significance Statement

Inhibition of hyaluronan synthesis induced by oral treatment with 4-methylumbelliferone disrupts perineuronal nets (PNNs) and diffuse extracellular matrix (dECM), reduces the astrocytic network, increases extracellular space (ECS) volume, and changes ECS geometry. The changes of diffusion barriers significantly affect diffusion parameters in the adult brain and spinal cord. Our findings suggest that disruption of ECM allows for more efficient transport of ions, neurotransmitters, and neuroactive substances in the ECS and thus ensures broader interneuronal communication by extrasynaptic transmission. Disruption of PNNs and an increase in ECS volume can result in enhanced cross talk between synapses, spillover of transmitters, formation of new synaptic contacts, and thus increased synaptic plasticity.

## Introduction

Neural extracellular matrix (ECM) is a network of macromolecules, glycosaminoglycans, proteoglycans, and glycoproteins, secreted by neurons and glia. Two ECM forms exist, aggregated perineuronal nets (PNNs), surrounding axons and some neurons, and a diffuse interstitial mesh (dECM) in the extracellular space (ECS; Yamaguchi, 2000; Dityatev et al., 2010). Changes in PNNs affect synaptic transmission, neuroplasticity, neural cell migration and differentiation, axonal growth, sprouting, and pathfinding (Kwok et al., 2010; Fawcett et al., 2019; Carulli et al., 2020). Dysregulation of PNNs has been described in amyotrophic lateral sclerosis (Forostyak et al., 2014), epilepsy (McRae et al., 2012), Alzheimer's disease (Scarlett et al., 2022), psychiatric diseases (Pantazopoulos and Berretta, 2016), and spinal cord injury (García-Alías et al., 2009; Wang et al., 2011). There has been an increasing effort to find ways to prevent the deleterious changes in the ECM accompanying various disorders and to enhance plasticity for the treatment of CNS diseases, e.g., by removing some components of the ECM (Pizzorusso et al., 2002; Galtrey et al., 2007; Wang and Fawcett, 2012; Hylin et al., 2013). Removal of PNNs has been usually performed using invasive methods, such as enzymatic degradation by bacterial enzyme chondroitinase ABC or hyaluronidase (Wang et al., 2011). An oral treatment with 4-methylumbelliferone (4-MU), a small-molecule regarded as an inhibitor of hyaluronan (HA) synthesis, has recently been shown to downregulate chondroitin sulfates (CSs) and disrupt PNNs (Kwok et al., 2011; Irvine et al., 2023; Stepankova et al., 2023). PNNs restrict transport of molecules near neuronal surface and synapses and act as a barrier between cells and ECS (Hanssen and Malthe-Sørenssen, 2022), and their removal affects synaptic transmission (Sorg et al., 2016). It is, however, unknown how the changes in PNNs and dECM modify extrasynaptic (volume) transmission mediated by diffusion of neuroactive substances in ECS (Fuxe and Agnati, 1991; Sykova, 1997; Zoli et al., 1999; Syková and Nicholson, 2008). ECS diffusion parameters are critically dependent on organization of ECM macromolecules and astrocytic network (Nicholson and Syková, 1998; Syková et al., 1998; Syková et al., 2002; Syková, 2004; Syková et al., 2005a; Syková and Nicholson, 2008; Bekku et al., 2010). Disruption of PNNs and dECM and changes in astrocyte morphology are accompanied by changes in ECS diffusion parameters during a variety of physiological and pathological states (Syková and Nicholson, 2008). Since the ECS is wider (Lehmenkühler et al., 1993), diffusion anisotropy is lower (Prokopová et al., 1997), and plasticity is higher during development than in adult and aged brain, it could be beneficial to modulate the ECS volume and diffusibility during treatment of neurological disorders in adulthood and aging. Diffusion of substances in the ECS depends on the size and charge of the diffusing substance and on the structure, architecture, and physicochemical properties of the ECS–nerve cell microenvironment (Nicholson and Phillips, 1981; Rice et al., 1993; Nicholson and Syková, 1998; Nicholson, 2022). In contrast to a free medium, diffusion in porous ECS is constrained by the restricted volume. The diffusion is hindered by the size of irregular pores, which sometimes have dead spaces, by the number of neuronal and glial processes, charged molecules, and the macromolecular composition of ECM. Diffusion in the ECS is constrained by the volume fraction *α* (*α* = ECS volume / total tissue volume; *α* ∼ 0.2 in the adult brain), by the tortuosity factor *λ* (*λ* = D/ADC, where D is the free diffusion coefficient and ADC is the apparent diffusion coefficient in the brain; *λ* ∼ 1.5–1.6 in the adult brain) reflecting a number of obstacles to diffusion in the ECS, and by the nonspecific cellular uptake *k*′. Therefore, the volume fraction and tortuosity which is often anisotropic significantly affect extrasynaptic movement of transmitters, synaptic “spillover,” synaptic cross talk, neuron–glia communication, spatial orientation of glial processes toward synapses, formation of new synapses, and, consequently, synaptic plasticity (Syková, 1997; Syková, 2004). Here we investigated the disruption of PNNs and dECM by oral delivery of 4-MU and its effects on the ECS volume and CNS diffusion rates in rats in vivo.

## Materials and Methods

### Animals and experimental design

Fifty-two 3-month-old female Wistar rats (Charles River Laboratories) were used in this study. Animals were housed under standard conditions with food and water *ad libitum* and a 12 h light/dark cycle. All experiments were conducted in accordance with EU directive 2010/63/EU and approved by animal ethics committees of the Institute of Scientific Instruments, Czech Academy of Sciences, and the Institute of Experimental Medicine, Czech Academy of Sciences. The animals were divided into two groups. In the first group, rats were fed standard diet (Sniff) containing 4-MU (DbPharma France) at a dose 1.25 g/kg/day and added chocolate flavor (4-MU diet). The second group of rats (*n* = 26), which served as controls, received a standard diet with added chocolate flavor (control diet). We noticed that the animals on the 4-MU diet consumed fewer pellets than the controls even with the chocolate flavor added. The animals were weighed weekly, and after 2 months, the control rats weighed 333.7 ± 9.73 g (*n* = 10), and the animals on the 4-MU diet weighed 274.6 ± 4.16 g (*p* < 0.001; *n* = 10).

For real-time tetramethylammonium iontophoretic (TMA-RTI) method measurements of the ECS diffusion parameters, we used six animals receiving the 4-MU diet and six control animals receiving the control diet for 3–6 months. In diffusion-weighted magnetic resonance imaging (DW-MRI) measurements, 10 control rats and 10 rats on the 4-MU diet were subjected to brain and spinal cord imaging after 2 months on their respective diets. Five animals from either group were then perfused for histology, and five animals from each group received standard diet for 2 additional months before a second imaging session was performed to evaluate a washout effect. To study the long-time effect of 4-MU, a new group of 20 animals underwent exposure to the 4-MU diet for 4 months (*n* = 5) and for 6 months (*n* = 5) before imaging and histology.

### Histology and immunohistochemistry

PNNs were visualized by the *Wisteria floribunda* agglutinin (WFA), a lectin which specifically labels *N*-acetylgalactosamine residues within the ECM of neurons. To assess the changes in the ECM composition, we measured the level of HA using HA-binding protein (HABP; AMS Biotechnology), CS proteoglycans (CSPGs) using CS56 antibody (anti-CSPG; Sigma-Aldrich), anti-aggrecan (anti-ACAN; Merck Millipore) antibody, or lectin WFA (Sigma-Aldrich) by densitometry measurement on brain sections. To visualize changes in astrocytes, we used immunohistochemistry staining for fibrillary acidic protein using anti-glial fibrillary acidic protein (GFAP) antibody (DAKO) and measured the level of staining by densitometry.

Paraformaldehyde-fixed brains were sectioned at 35 µm using a cryostat. The sections were labeled with biotinylated WFA (1:150), HABP (1:150), CS56 (1:300), anti-ACAN antibody (1:300), or anti-GFAP antibody (1:600) overnight at 4°C, then washed three times with 1× PBS-0.3% Triton X-100, and labeled with streptavidin Alexa Fluor 488 or Alexa Fluor 568 antibody (1:500), anti-mouse IgM Alexa Fluor 405 or Alexa Fluor 488 antibodies (1:500), or anti-rabbit Alexa Fluor 647 for 3 h at room temperature. The sections were washed again twice in 1× PBS-0.3% Triton X-100 and then once with Tris nonsaline, before mounting on glass slides with FluoroSave and imaging with the Axioscan Z.1 Slidescanner (Carl Zeiss). All images were taken with the same settings using the Slidescanner. Densitometry was then performed using Fiji image processing package (www.fiji.sc).

Morphological characteristics of the astrocytic cell population were assessed by a blinded observer using Fiji image processing package. Astrocytes from Layer V of the primary somatosensory cortex, identifiable by the GFAP signal, were randomly selected. Two *z*-stack images (15 planes with a step size of 1 μm) were captured from both the left and right hemispheres. Each *z*-stack image was processed semiautomatically using the same macro in Fiji. The processing sequence began with background subtraction, followed by the application of an unsharp mask and despeckle filter to enhance the GFAP signal and reduce noise. Autothresholding was then performed to generate binary masks for each image. These binary masks were refined using the BioVoxxel Toolbox plug-in to connect cell processes. Each thresholded image was carefully inspected for accurate reconstruction, with manual adjustments made using the paintbrush tool to merge any remaining cell ramifications. A particle analysis function was applied to “clean” the final binary masks, ensuring that only cells of interest were included for further analysis. Astrocytic binary masks were randomly selected using the ROI Manager tool in Fiji. Shape descriptor parameters, including area, perimeter, aspect ratio, circularity, and solidity, were calculated for each selected astrocyte, following previously described methods (Baldwin et al., 2024). At least four cells per slice were quantified, resulting in ∼33 cells per animal, with at least 100 cells analyzed per condition. The same binary masks were also used for skeleton analysis using the Analyze Skeleton plug-in in Fiji to obtain a number of branches and junctions, average and maximum branch lengths, triple points, quadruple points junction voxels, end-point voxels, and slab voxels.

### In vivo TMA-RTI method

Control rats (*n* = 12) and rats on the 4-MU diet (*n* = 6) were anesthetized with inhalation of isoflurane (Isoflurin, Vetpharma Animal Health) and a small craniotomy was made above the somatosensory cortex. The real-time iontophoretic method is the only method that can be used to determine the absolute values of all three ECS diffusion parameters, i.e., the volume fraction *α*, the tortuosity *λ*, and the nonspecific uptake *k*′ in the nervous tissue (Nicholson and Phillips, 1981). Ion-selective microelectrodes (ISM) are used to measure the diffusion of ions for which the cell membranes are relatively impermeable, e.g., tetraethylammonium (TMA^+^) ions. Here, TMA^+^ ions were administered by an iontophoretic electrode aligned parallel to a double-barreled ISM at a fixed distance (Svoboda and Syková, 1991). An electrode array was made by gluing together an iontophoretic pipette and a TMA^+^–ISM with a tip separation of 80–200 µm (Fig. 3*A*, scheme). TMA^+^ was released from the iontophoretic pipette into the ECS by applying a continuous positive bias current of 20 nA from a constant-current source. To obtain a diffusion curve, we applied a current step of +80 nA with a duration of 40–80 s. The signal from double barrel TMA^+^–ISM was connected to a dual-channel preamplifier to subtract the DC potential from the reference barrel from the signal of the ion-sensing barrel and then further amplified. The TMA^+^–ISM diffusion curves were analyzed by programs VOLTORO (kindly provided by C. Nicholson) and WANDA written in MATLAB language (Hrabetova and Nicholson, 2007). The values of *α*, ADC_TMA_, *λ*, and *k*′ were extracted by a nonlinear curve-fitting simplex algorithm operating on the diffusion curve described by a modified diffusion equation as described below, which represents the behavior of TMA^+^ in ECS assuming that it spreads out with spherical symmetry when the iontophoresis current is applied for a duration S. The equation governing diffusion in the brain tissue is as follows:

C(t)=G(t)fortherisingphaseofthecurve(t<S)

C(t)=G(t)−G(t−S)forthefallingphaseofthecurve(t≥S),

where *C*(*t*) is the concentration of the ion at time *t* and distance *r* and function G(τ) is defined by Nicholson and Phillips (1981) as follows:

$$\begin{array}{rl} G(\tau ) & =\frac{Q\lambda^{2}}{8\pi \;D\;\alpha \;r}\{\exp\;\left(r\lambda \sqrt{\frac{k^{\prime }}{D}}\right)\;erfc\left(\frac{r\lambda }{2\sqrt{D\tau }}+\sqrt{k^{\prime }\tau }\right)\\ & \;+\exp\;\left(-r\lambda \sqrt{\frac{k^{\prime }}{D}}\right)erfc\left(\frac{r\lambda }{2\sqrt{D\tau }}-\sqrt{k^{\prime }\tau }\right)\}, \end{array}$$

where *Q* = *In*/*zF* is the quantity of TMA^+^ delivered to the tissue per second, *I* is the step increase in current applied to the iontophoresis electrode, *n* is the transport number, *z* is the number of charges associated with the substance released by iontophoresis (+1 for TMA^+^), and *F* is the Faraday's electrochemical equivalent. The function “erfc” is the complementary error function. Electrodes were first calibrated in an experimental medium (agar), where, by definition, *α* = 1 = *λ* and *k'* = 0 and the parameters *n* and *D* were extracted by curve fitting. Knowing *n* and *D*, the parameters *α*, *λ*, and *k*' were obtained when the experiment was repeated in the brain tissue. To be able to evaluate even subtle changes in ECS diffusion parameters, the same TMA^+^–ISM was always used in two animals, one control animal and one 4-MU–treated animal.

### MRI experiments

MRI experiments were performed on a Bruker BioSpin 9.4 T scanner (Bruker BioSpin 94/30 Avance III USR HD, Germany). A quadrature volume transmit and a four-channel surface receive radiofrequency coils were used. Rats were anesthetized (1 ml/min oxygen with 2% isoflurane) and placed onto a heated bed during the experiment. Body temperature and breathing were monitored continuously throughout the measurement. The same experimental protocol was used for all animals and all measurements.

### Anatomical T2 images

Rat brains were imaged using T2-weighted multislice multiecho (MSME) with TR = 3,000 ms, 12 TEs equally distributed from 10 to 120 ms, 22 axial slices with FOV 3.5 × 3.5 cm^2^, image and acquisition matrix 256 × 256 (with 32 zero-filled *k*-space rows), and slice thickness 1 mm. Besides anatomical images, T2 maps were calculated from these datasets in ParaVision software v. 7.0 (Bruker, Biospin MRI).

### DW-MRI

Diffusion tensor data (DTI) were acquired with a spin-echo echoplanar imaging pulse sequence. Diffusion-weighting gradient schemes with 60 noncollinear directions were used. The acquisition parameters were as follows: TR, 3,500 ms; TE, 22.9 ms; 35 axial slices (22 slices at the same positions as in T2 MSME) with 1 mm slice thickness and FOV 3.5 × 3.5 cm^2^, with matrix 128 × 128 voxels with GRAPPA acceleration; four segments; *b* value of 800 s/mm^2^; and diffusion time Δ = 11 ms. After 1 more week, the animals underwent a DTI measurement of the thoracic spine. In these measurements, motion artifacts were avoided by triggering to breathing and ECG of the animal, and diffusion-weighting gradient schemes with 30 noncollinear directions were used. Twelve contiguous slices with slice thickness of 2 mm and FOV 3.5 × 3.5 cm^2^ were measured in eight segments and full matrix size of 128 × 128 voxels, applying TR, 2,500 ms; TE, 24.3 ms; *b* = 800 s/mm^2^; and diffusion time Δ = 11 ms. One-slice saturation was used to eliminate the signal from the heart and the thoracic space. The DTI datasets were spatially registered, and diffusion images with artifacts were removed from calculations. Mean diffusivity (MD) and fractional anisotropy (FA) values were then calculated from the DTI datasets in DSI studio (https://dsi-studio.labsolver.org).

### Proton magnetic resonance spectroscopy (^1^H MRS)

Proton MRS single-voxel measurements were performed in two regions, the right dorsal hippocampus and the right somatosensory cortex. The voxel positions in both regions based on T1-weighted anatomical reference images are depicted in Figure 6 where the red lines represent voxel borders. T1-weighted images in axial, sagittal, and coronal orientations were measured using the RARE sequence (TR, 600 ms; TE_eff_, 21.4 ms; TE, 7.1 ms; RARE-factor 6; matrix 192 × 192 voxels, FOV 3.5 × 3.5 cm^2^, 10 slices, 0.8 mm slice thickness). ^1^H MRS acquisition was performed using the PRESS sequence (voxel volume 5.58 mm^3^ for the hippocampus and 7.84 mm^3^ for the cortex, spectral width 4,401.41 Hz; TE,16.5 ms with TE1, 8.85 ms, and TE2, 7.65 ms; TR, 2,500 ms; 256 averages) VAPOR water suppression and outer volume suppression, and MapShim shimming based on a B0 map within the shimming volume (Fig. 6*A*,*B*, green lines). MRS data were collected and processed as described previously (Pavlova et al., 2023). The processing was done using the jMRUI software (Stefan et al., 2009) with its automated QUEST-MM algorithm (Starcukova et al., 2023), using a basis set calculated with jMRUI's simulator NMRScopeB (Starcuk and Starcukova, 2017) applying the same parameters as the in vivo experiments to the detected metabolites. The investigated metabolites were as follows: mIns, *myo*-inositol; tCr, total creatine (tCr = Cr + PCr; Cr, creatine; PCr, phosphocreatine), Glx (Glx = Glu + Gln; Glu, glutamate; Gln, glutamine); Tau, taurine; Cho, choline; and NAA, *N*-acetyl aspartate.

### Statistical analysis

Data are presented as mean ± SEM in column bar graphs and as medians and quartiles in box plots with whiskers indicating the range of values. Differences between corresponding groups of animals (control groups vs groups on the 4-MU diet) were evaluated using unpaired two-tailed *t* tests. *p* < 0.05 were considered statistically significant. All *p* values are presented without adjustment for multiple comparisons.

## Results

### 4-MU diet downregulates ECM composition and affects astrocytes

**Figure 1.**
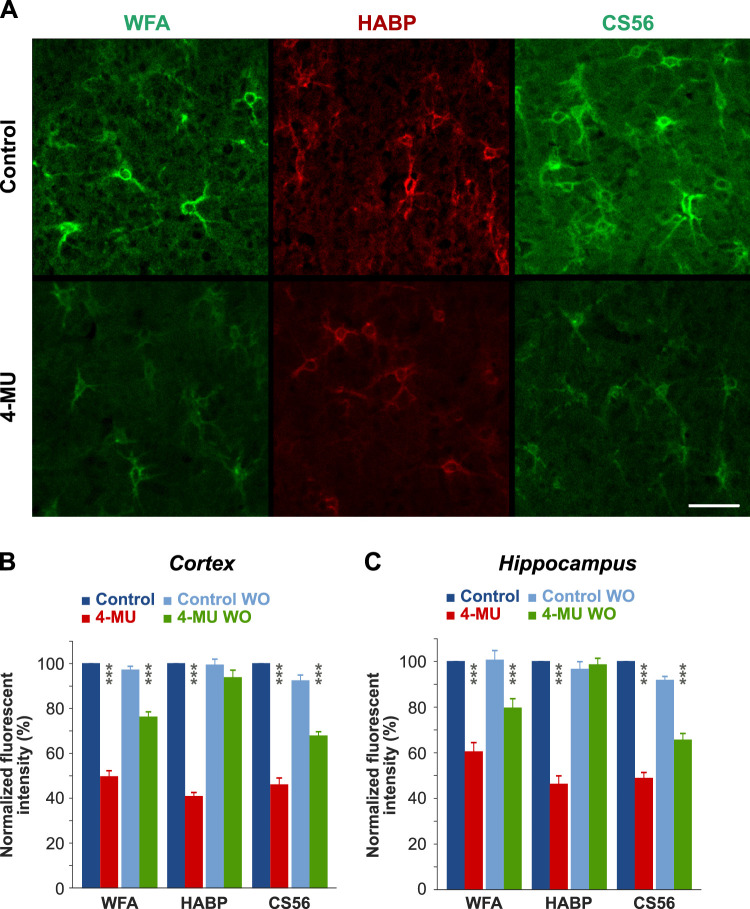
Immunohistochemistry revealing the downregulation of PNNs and CS after 4-MU administration. ***A***, Representative images of somatosensory cortical sections (Layers III–IV) stained for (left) WFA, (middle) HABP, and (right) CS56 antibody for HA and CS show weaker staining intensity after 4-MU treatment. Scale bar, 50 μm. ***B*, *C***, Staining intensities of WFA, HABP, and CS56 were measured by densitometry in cortical (***B***) and hippocampal sections (***C***) in control animals after 2 months (Control 2m, blue), control animals after 4 months (Control 4m, light blue), 4-MU–treated animals after 2 months (4-MU 2m, orange), and 4-MU animals after “washout” (4-MU 2m WO, light orange); *n* = 5 in each group. ***p < 0.001.

To evaluate the changes in the ECM composition after the 4-MU diet, we assessed the changes in the composition of PNNs and dECM by immunohistochemistry. Figure 1*A* shows that PNNs in Layer V of the somatosensory cortex significantly decreased in rats exposed to the 4-MU diet as demonstrated by immunofluorescence staining for WFA, HABP, and CS56 antibody for PNN, HA, and CSs. To show changes in the dECM, we measured the levels of WFA, HA (using HABP), and CSs (using CS56 antibody) by densitometry measurements on brain sections. In Figure 1, B and *C*, we present a decrease in fluorescence intensity after 2 months of the 4-MU diet in the somatosensory cortex and in the CA1 region of the hippocampus as well as values measured 2 months after the rats returned to the control diet (washout effect). In the cortex, the normalized fluorescence intensity of staining for WFA, HABP, and CS56 decreased on average by 50.4%, 59.2%, and 54.0%, respectively, in the CA1 region of the hippocampus by 40.6%, 53.8%, and 51.2%, respectively.

**Figure 2.**
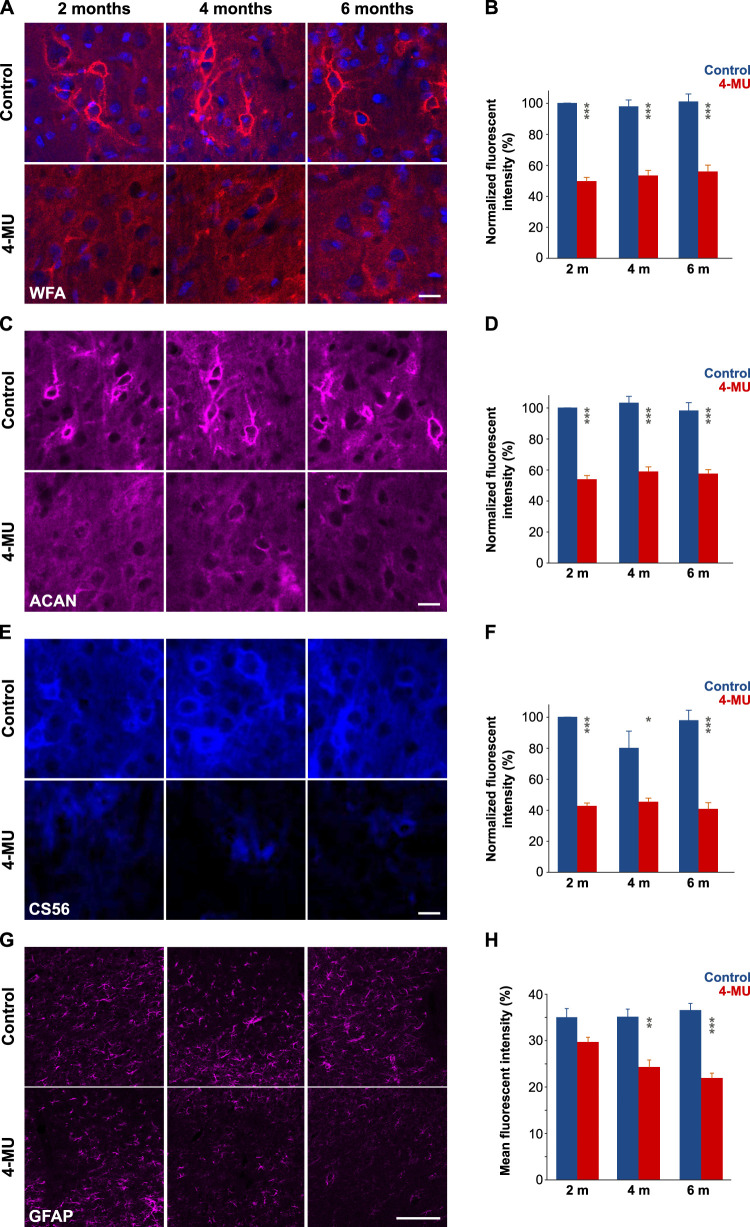
Immunohistochemistry revealing a persistent downregulation of PNNs, CS, and GFAP after 4-MU administration in Layer V of the rat somatosensory cortex. Panels ***A***, ***C***, ***E***, and ***G*** show typical expression of WFA, ACAN, CS56, and GFAP, respectively, in control animals on a control diet and in animals treated with 4-MU. Scale bars, 50 μm. ***B*, *D*, *F*, *H***, Densitometry measurements show a persistent decrease in the corresponding fluorescence intensity at 2, 4, and 6 months of 4-MU treatment; mean ± SEM; *n* = 3–5 in each group, **p < 0.01; ***p < 0.001.

PNNs and dECM were consistently downregulated after 2, 4, and 6 months of 4-MU diet as manifested by decrease in the fluorescence intensities of staining for WFA, ACAN, and CS56 in the somatosensory cortex (Fig. 2*A*,*C*,*E*). Densitometry measurements in the somatosensory cortex showed a persistent decrease in normalized fluorescence intensity in the 4-MU–treated animals at 2, 4, and 6 months compared with the controls. WFA intensity remained decreased (on average) by 50.4%, 44.6%, and 45.2% at 2, 4, and 6 months of the 4-MU diet, respectively (Fig. 2*B*). Similarly, ACAN intensity decreased by 46.2%, 44.4%, and 40.8% (Fig. 2*D*), and CS56 intensity decreased by 57.4%, 54.8%, and 57.2% (Fig. 2*F*).

Immunohistochemistry of GFAP revealed a statistically significant downregulation of GFAP expression in astrocytes in animals at 2, 4, and 6 months on the 4-MU diet. Figure 2*G* shows representative images of GFAP staining in control animals and in animals exposed to 4-MU diet. Figure 2*H* presents densitometry measurements expressed as mean fluorescence intensity of GFAP staining, decrease in fluorescence intensity by 5.2% after 2 months, by 10.8% (*p* < 0.01) after 4 months, and by 14.6% (*p* < 0.001) after 6 months of the 4-MU diet, suggesting a progressive change in the structure of the astrocytic network.

**Figure 3.**
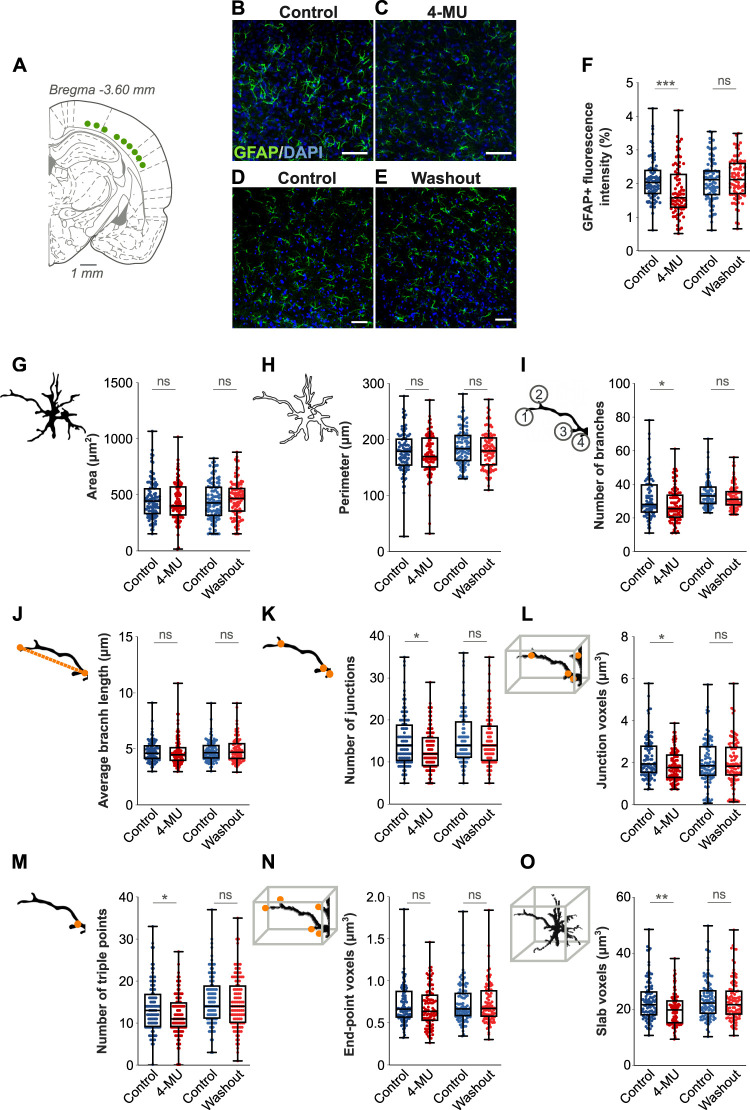
Morphometric analysis of astrocytes. ***A***, Schematic representation of the coronal brain section showing the region of interest in the Layer V of the primary somatosensory cortex. ***B*, *C***, Representative immunofluorescence images from control and 4-MU–treated rats, respectively. ***D*,** ***E***, Representative immunofluorescence images from control and 4-MU washout rats, respectively; GFAP, green; DAPI, blue. ***F***, Densitometry measurements showed a decrease in the fluorescence intensity at 2 months of 4-MU treatment, with subsequent recovery of fluorescence intensity following a 2 month washout period. ***G*, *H***, Quantification of astrocytic cell area and perimeter showed no statistically significant difference between the control and 4-MU–treated groups. ***I***, Skeleton analysis revealed a significant reduction in the number of astrocytic branches in 4-MU–treated animals compared with controls, indicating decreased complexity of astrocytic processes. ***J***, No significant difference was found in the average branch length between the control and 4-MU groups. ***K*, *L***, Quantification of junctions and junction voxels showed significant reduction in 4-MU–treated animals, suggesting reduced astrocytic connectivity. ***M***, Quantification of triple points showed significant reduction in 4-MU–treated animals. ***N***, End-point voxels, representing terminal points of astrocytic processes, did not differ significantly between control and 4-MU–treated animals. ***O***, Skeletal analysis further revealed a significant reduction in slab voxels of astrocytes in 4-MU–treated animals when compared with controls, indicating a decrease in astrocyte volume. Scale bars, 50 µm; boxes indicate medians and quartiles; whiskers indicate the range of values; *n* = 3 rats; 8 slices/animal; 4–5 astrocytes/slice; ∼100 astrocytes/condition in panels ***F***–***O***; ns = not significant; *p < 0.05; **p < 0.01; ***p < 0.001.

We performed detailed morphometric analysis of astrocytes in Layer V of the somatosensory cortex after 2 months of the 4-MU diet as well as 2 months after the rats returned to the control diet (washout rats). Figure 3*B–E* shows representative immunofluorescent images of GFAP staining in 4-MU–treated and washout rats, alongside their age-matched controls. Densitometry measurements showed a statistically significant decrease in fluorescence intensity (*p* = 0.0005; *p* = 0.5668; Fig. 3*F*). Analysis of size and shape descriptors revealed no statistically significant differences in area (*p* = 0.5531; Fig. 3*G*), perimeter (*p* = 0.548; Fig. 3*H*), circularity (*p* = 0.269), aspect ratio (*p* = 0.188), and solidity (*p* = 0.497) between animals on the 4-MU diet for 2 months and controls. However, skeleton analysis indicated statistically significant reductions in the number of branches (*p* = 0.027; Fig. 3*I*) and junctions (*p* = 0.021; Fig. 3*K*) in the 4-MU group compared with those in controls. Additionally, measurements related to astrocyte volume and connectivity, including junction voxels (*p* = 0.0162; Fig. 3*L*), triple points (*p* = 0.032; Fig. 3*M*), and slab voxels (*p* = 0.002; Fig. 3*O*), showed statistically significant decreases. In contrast, other skeletal parameters such as end-point voxels (*p* = 0.113; Fig. 3*N*), average branch length (*p* = 0.293; Fig. 3*J*), maximum branch length (*p* = 0.552), and quadruple points (*p* = 0.287) did not exhibit statistically significant changes.

Further analysis in the washout and corresponding control groups demonstrated no statistically significant differences in astrocytic shape and size descriptors, including area (*p* = 0.783; Fig. 3*G*), perimeter (*p* = 0.616; Fig. 3*H*), circularity (*p* = 0.170), aspect ratio (*p* = 0.432), or solidity (*p* = 0.930). Skeleton analysis also indicated no statistically significant differences in the number of branches (*p* = 0.709; Fig. 3*I*), average branch length (*p* = 0.865; Fig. 3*J*), number of junctions (*p* = 0.955; Fig. 3*K*), junction voxels (*p* = 0.998; Fig. 3*L*), triple points (*p* = 0.746; Fig. 3*M*), end-point voxels (*p* = 0.973; Fig. 3*N*), and slab voxels (*p* = 0.976; Fig. 3*O*). Overall, these findings suggest that while the area and perimeter of astrocytes remained relatively stable, their complexity and connectivity were reduced in rats on 4-MU diet. This effect was transient, with astrocytes recovering after rats returned to control diet.

### 4-MU diet increases ECS volume as measured by the RTI method

**Figure 4.**
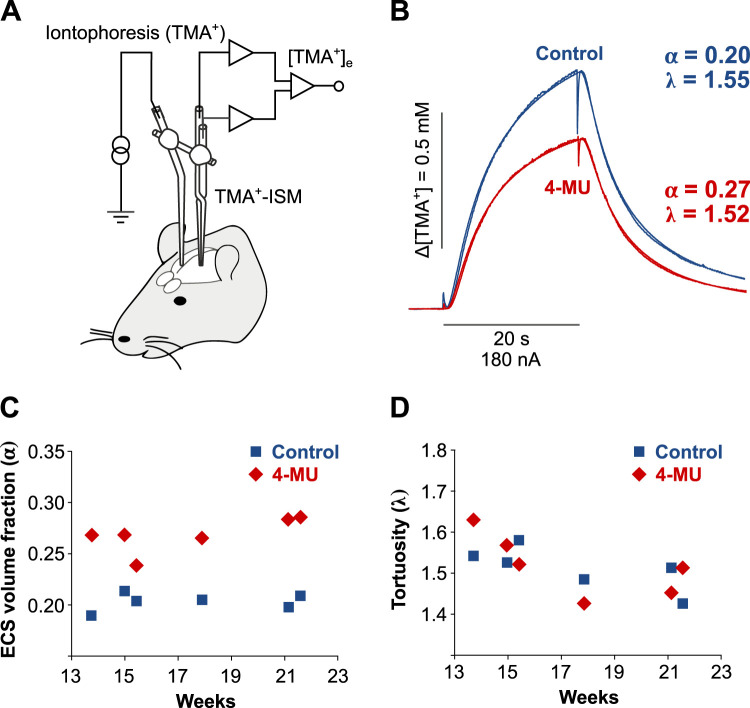
Changes in ECM volume and geometry after 4-MU administration were estimated using real-time iontophoretic TMA^+^ method for measurement of ECS diffusion parameters. ***A***, Schematic diagram of experimental arrangement with a TMA^+^ ISM array. ***B***, Typical pairs of diffusion curves recorded in Layer V of the somatosensory cortex of a control and a 4-MU–treated rat. ***C*, *D***, Absolute values of volume fraction α and tortuosity *λ* in control rats (*n* = 6) and 4-MU–treated rats (*n* = 6) were recorded between 13 and 23 weeks. The values shown at each timepoint are average values computed from two diffusion curves recorded with the same microelectrode array in one control and one 4-MU–treated rat in cortical Layer V.

To study the possible effect of the 4-MU diet on disorganization of ECM, we estimated changes in ECS volume and geometry (Fig. 4*A*) by means of TMA^+^–RTI in vivo. Diffusion measurements were performed between 3 and 5 months after the start of control or the 4-MU diet. From each animal, we recorded at least three TMA^+^ diffusion curves in Layers III, IV, V, and VI of the somatosensory cortex, where diffusion is isotropic. Figure 4*B* shows the typical diffusion curves recorded in cortical Layer V in a control rat and in a 4-MU–treated rat with the calculated values of *α* and λ (note the smooth calculated curve superimposed on the actual recorded curve). Figure 4*C* shows that the volume fraction α was consistently higher in all animals treated for 3–5 months with the 4-MU diet. The values calculated from six animals and two stable diffusion curves in cortical Layer V were *α* = 0.27 ± 0.008 (*n* = 12) in the 4-MU–treated rats compared with *α* = 0.20 ± 0.003 (*n* = 12; *p* < 0.001) in the control rats. The tortuosity values (*λ*) were not significantly different in the control rats (*λ* = 1.53 ± 0.023; *n* = 12) and in the 4-MU–treated animals (*λ* = 1.52 ± 0.024; *n* = 12; Fig. 4*D*). There was also no change in the nonspecific uptake: *k*′ = 0.0089 ± 0.0012 in the control rats and *k*′ = 0.0056 ± 0.001 in the 4-MU–treated rats. We conclude that the 4-MU diet led to significant increase in the ECS volume.

### 4-MU diet decreases MD and diffusion anisotropy

**Figure 5.**
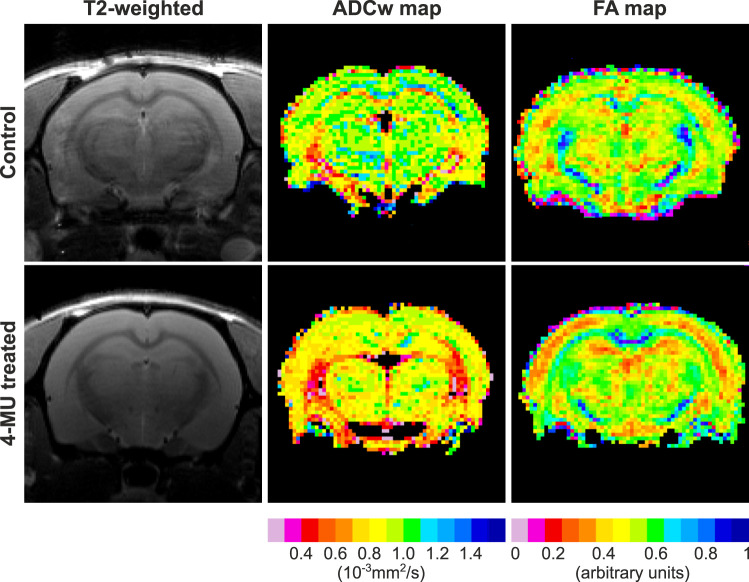
Representative T2-weighted sagittal images and color maps of ADCw and FA in control and 4-MU–treated rats (field of view 3.5 × 3.5 cm^2^; matrix size 128 × 128 voxels). Note the lower ADCw and FA values throughout the whole slice in a 4-MU–treated rat group compared with the slice in a control rat.

**Figure 6.**
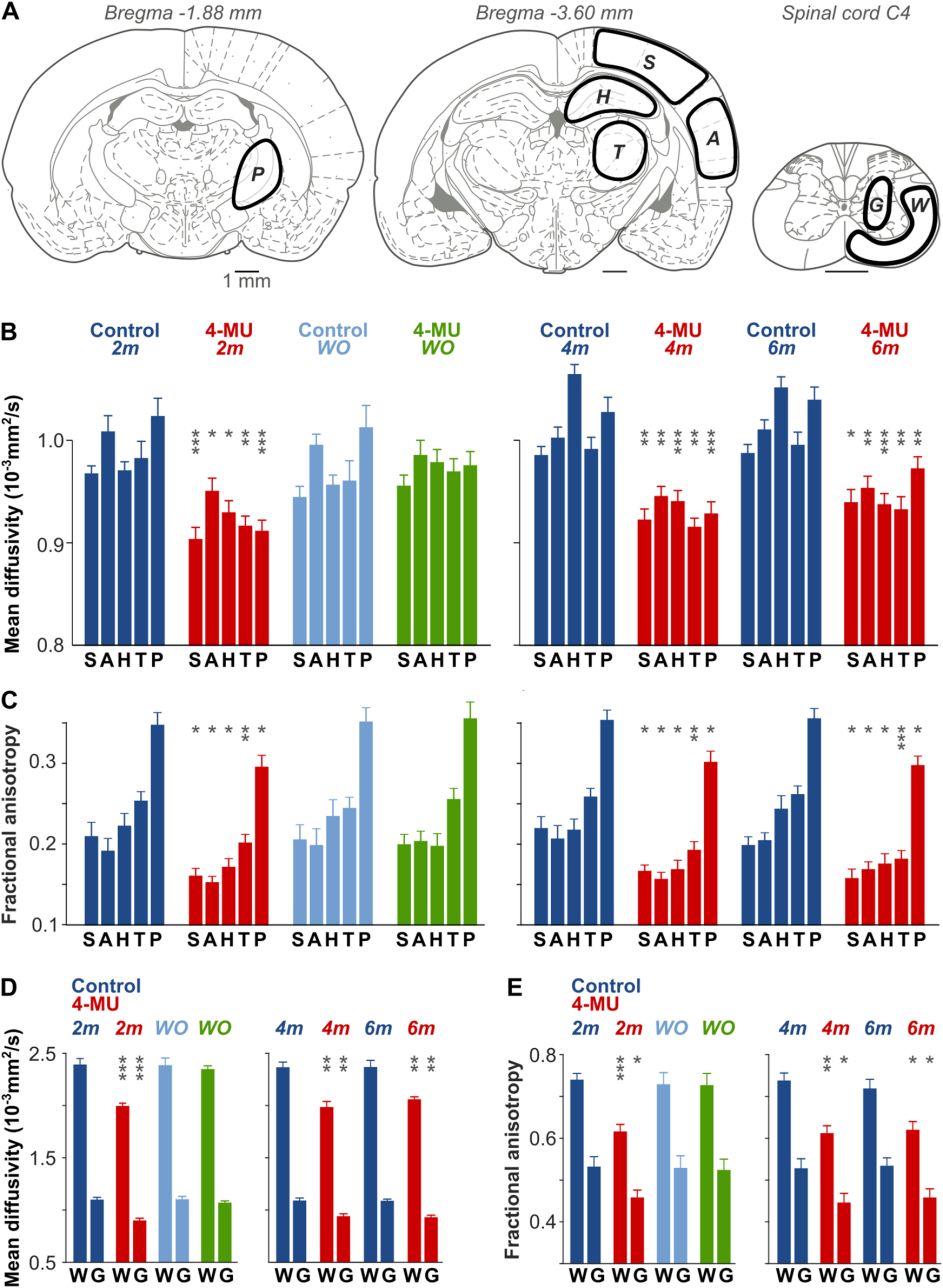
MD and FA decreased in 4-MU–treated animals. ***A***, MD and FA values were examined in five brain areas and spinal cord. *S*, primary somatosensory cortex; *A*, auditory cortex; *H*, hippocampus; *T*, thalamus; *P*, pallidum; *G*, spinal cord gray matter; *W*, spinal cord white matter (Paxinos and Watson, 2013). ***B*, *D***, MD values were lower in five brain areas (***B***) and spinal cord (***D***) in animals exposed to 4-MU for 2, 4, and 6 months. ***C*, *E***, FA values were also lower after 2, 4, and 6 months of 4-MU diet in the brain (***C***) and spinal cord (***E***). Note that MD and FA values returned to normal values after the animals were exposed to control diet (washout), showing that the changes in diffusivity were transient. Bars represent mean ± SEM in each group; asterisks denote *p* values for differences between the means of 4-MU groups and their corresponding control groups; *p < 0.05; **p < 0.01; ***p < 0.001.

In vivo DW-MRI was used to study changes in MD and FA in rats after the 4-MU diet. Figure 5 presents typical T2-weighted axial images, maps of the apparent diffusion coefficient of water (ADCw) and maps of FA in axial scans (field view 3.5 × 3.5 cm^2^; matrix size 128 × 128 voxels) of a control rat and a rat treated for 2 months with the 4-MU diet. Diffusion in rat brains was not homogeneous; ADCw and FA were lower throughout the axial slice in the 4-MU–treated rat in comparison with the control rat (Fig. 5). We studied the effect of 4-MU after 2, 4, and 6 months on the 4-MU diet in five brain areas and in the spinal cord gray and white matter (Fig. 6*A*). We found a persistent and statistically significant decrease of both MD and FA in the somatosensory cortex (S), auditory cortex (A), hippocampus (H), thalamus (T), and pallidum (P) as well as in gray (G) and white matter (W) of the spinal cord (Fig. 6*B*,*C*,*D*,*E*). After measurements at the 2 month timepoint, a group of 4-MU–treated rats returned to a control diet, and the changes were studied again after 2 months (washout effect). The changes in MD and FA evoked by the 4-MU diet were transient; there were no statistically significant differences observed between the rats returned to the control diet and the respective controls (Fig. 6*B*,*C*,*D*,*E*). We conclude that the changes in the brain microstructure evoked by 4-MU altered water diffusibility in the brain and spinal cord.

### Effect of 4-MU diet on proton spectra

**Figure 7.**
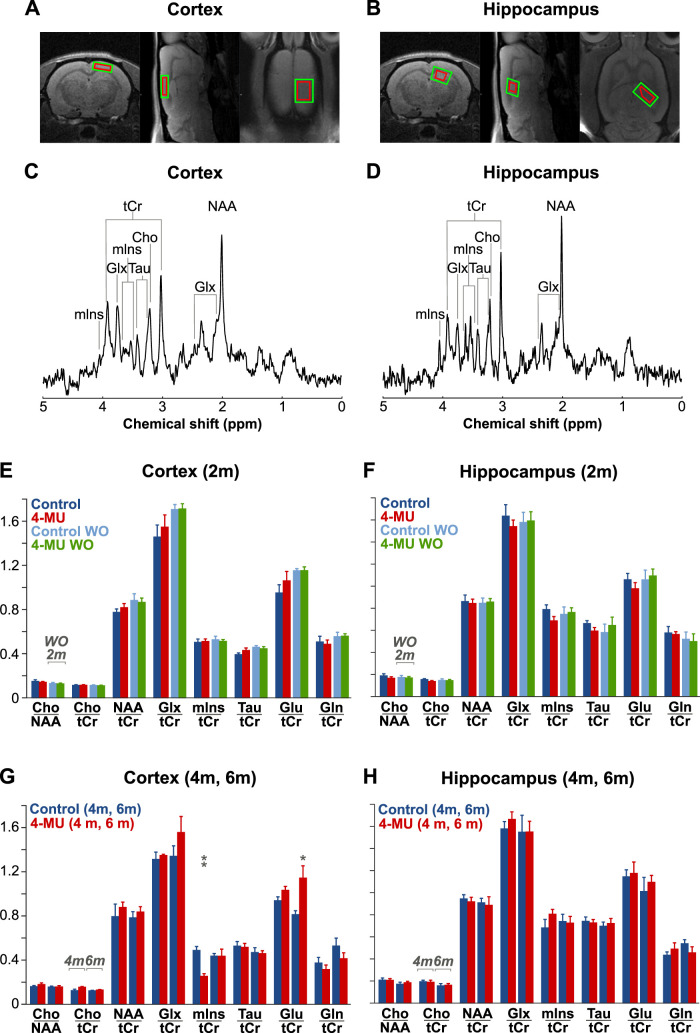
Exposure to 4-MU diet did not affect concentrations of important metabolites. ***A*, *B***, Voxel positions (red cuboid) in MRS experiments based on T1-weighted anatomical reference image in the right somatosensory cortex (***A***) and the right dorsal hippocampus (***B***). Representative spectra from the cortical (***C***) and hippocampal (***D***) voxels with labeled peaks of detected metabolites: mIns, *myo*-inositol; tCr, total creatine (tCr = Cr + PCr; Cr, creatine; PCr, phosphocreatine); Glx, glutamate–glutamine (Glx = Glu + Gln; Glu, glutamate; Gln, glutamine); Tau, taurine; Cho, choline; NAA, *N*-acetyl aspartate (***E*, *F*, *G*, *H***). Metabolite concentration ratios in the somatosensory cortex (***E*, *G***) and the hippocampus (***F*, *H***) detected: (***E*, *F***) after 2 months on the diet in control rats (navy) and in 4-MU–treated rats (red) and after 2 following months of washout with control diet in the same control (azure) and 4-MU–treated rats (green); (***G*, *H***) after 4 and 6 months on the diet in control (navy) and 4-MU–treated (red) rats. Each bar represents a metabolite ratio mean ± SEM; *n* = 3–10 in each group; asterisks denote *p* values for differences between the means of 4-MU groups and their corresponding control groups with *p < 0.05; **p < 0.01.

To see whether the 4-MU diet substantially influences neural cells, we studied proton spectra in the cortex and the hippocampus (Fig. 7*A*,*B*) in control and 4-MU–treated rats. Representative proton spectra in the cortex (Fig. 7*C*) and hippocampus (Fig. 7*D*) show the peaks of the investigated metabolites: creatine and phosphocreatine, *myo*-inositol, taurine, choline, glutamate and glutamine, and *N*-acetyl aspartate. Figure 7, *E* and *F*, compares the concentration ratios in the control and the 4-MU–treated rats as measured in the cortex (Fig. 7*E*) and hippocampus (Fig. 7*F*). We found no statistically significant differences between the control (*n* = 10) and the 4-MU–treated groups (*n* = 10) after 2 months of the 4-MU diet. In the cortex, we found a statistically significantly lower mIns/tCr ratio after 4 months, which were normalized after 6 months of 4-MU diet, and higher Glu/tCr ratio after 6 months of the 4-MU diet rats (Fig. 7*G*) compared with controls. There were no statistically significant differences detected in the hippocampus (Fig. 7*H*). We show that the disruption of PNNs and dECM after the 4-MU diet does not affect tissue concentrations of most of the important metabolites and that the distinct observed changes in mIns/tCr and in Glu/tCr might reflect changes in astrocytes.

## Discussion

**Figure 8.**
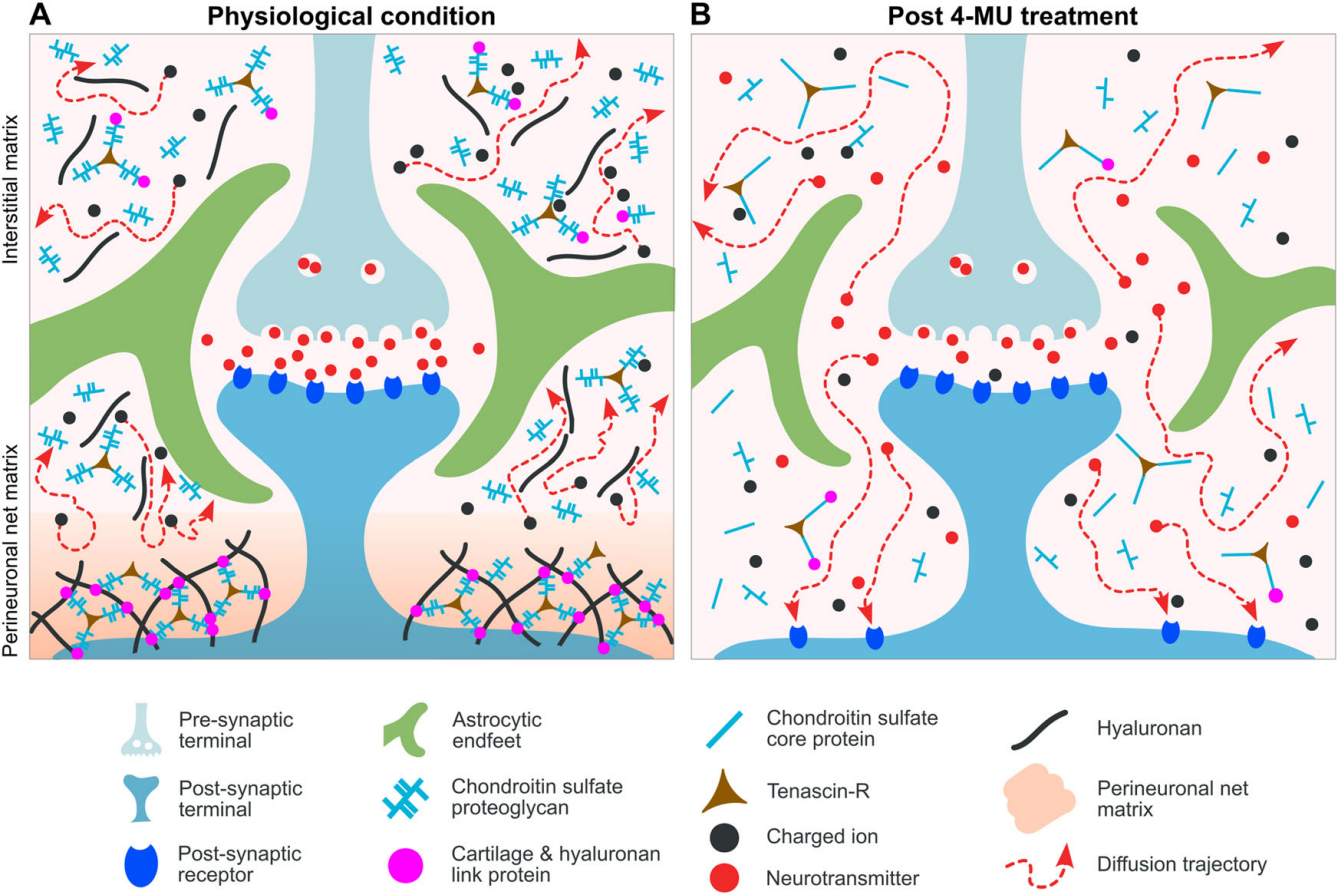
Schematic diagram presenting structural and diffusion changes before (***A***) and after (***B***) the disorganization of ECM by 4-MU. ***A***, Under physiological conditions, the diffusion of ions and neuroactive molecules through ECS is limited by dECM, PNNs, and glial processes indicated by red lines with arrows. PNNs form a diffusion barrier; transmitters cannot leave the synaptic cleft and reach extrasynaptic receptors. ***B***, After blocking HA synthase, HA and CS proteoglycans are disrupted, PNN matrix is removed, the mesh-like structure of ECM is less dense, ECS volume increases, and the tissue is more permeable for diffusion (indicated by red arrows). The space between thinner astrocyte end feet and synapse is increased, and the diffusion of transmitters is no more limited to the synaptic cleft. Transmitters can escape from the synapse (spillover effect), reach extrasynaptic receptors that are not covered by PNNs, and diffuse through ECS to nearby membranes and synapses (cross talk between synapses).

HA and CSPGs are major components of the neural ECM and core constituents of the organization of the ECM network (Yamaguchi, 2000). Its disruption can cause dramatic changes in neuron to neuron and neuron to glia interaction and function. Here we showed that oral treatment of rats with 4-MU, an inhibitor of HA and CS synthesis, resulted in a decrease in PNNs and dECM components as demonstrated by downregulation of staining for PNNs, HA, and CSPGs (Figs. 1 and 2). This downregulation in ECM was accompanied by a decrease in GFAP staining, complexity, and overall volume of astrocytes (Fig. 3). Importantly, this effect was transient; astrocyte changes returned to control values after the animals were treated with control diet. A summary schematic depicting dysregulation in PNNs, dECM, and retraction and shrinkage of astrocytic processes is shown in Figure 8. Neurodegenerative disorders, CNS injury, aging, lactation, and others are associated with astrogliosis; changes in astrocytic network and astrocytes and PNNs interact abnormally (Syková, 1997; Syková, 2004; Syková and Nicholson, 2008). Indeed, during brain inflammation the degradation of PNNs which are attached to astrocytic processes via HA–CD44 complex leads to retraction of astrocytic processes from synapses (Woo and Sontheimer, 2023). Our data suggest that inhibition of HA and CS synthesis by a 4-MU diet results in degradation of PNNs and remodeling of the astrocytic network. The residue of HA chains, together with lack of CSs for cross-linking, leads to an increase in the ECS volume (Fig. 8*A*,*B*). The potential reason for the increase in ECS could therefore be the lack of CSs in PNNs and dECM, which leads to disassembly of the aggregated PNN matrix. HA has a high binding capacity for water; therefore, it occupies a large molecular domain in extracellular fluid. Due to the hydrophilic properties of HA, HA chains may also increase its hydrodynamic volume and thus occupy larger ECS (Toole, 2004). At the same time, the increase in HA viscosity due to the absorbed water may also partly explain the observed reduced water diffusivity. Disruption of HA chains and CSPGs after 4-MU diet can therefore lead to enhanced extrasynaptic transmission by diffusion of ions, neurotransmitters, and other neuroactive substances to extrasynaptic receptors and nearby neurons (Fig. 8*B*).

4-MU–evoked changes in tissue microstructure should undoubtedly affect diffusion of water in the nervous tissue. In our in vivo diffusion analyses, we found that despite the greatly enlarged ECS volume *α* by ∼35%, the overall extracellular diffusivity measured by DW-MRI as ADCw was significantly reduced in all studied brain areas as well as in spinal cord gray and white matter. This shows that diffusion of water differs from diffusion of small cations TMA^+^. It should be kept in mind that diffusion of TMA^+^ to which cell membranes are impermeable is purely extracellular, while the diffusion of water is intracellular as well as extracellular. However, the advantage of DW-MRI is that it reveals changes in MD and FA in the entire brain and spinal cord. The observed changes reflect the fact that diffusion rates measured by RTI and DW-MRI describe particle mobility at different spatial and temporal scales. Ionic diffusion in the ECS occurs at several levels; at a nanoscale level between cell soma and PNNs, at 0,5–3 µm level between nearby cells and dendritic spines; and at 100–300 µm level in interneuronal and neuron–glia communication. The RTI method measures the diffusion of small ions TMA^+^ at space widths of 100–200 µm and thus cannot characterize diffusion parameters between neuronal surface and PNNs. Soria et al. (2020) studied diffusion at the nanoscale level using nanotube tracking in synucleinopathy-induced degradation of the HA matrix. They found that α-syn–induced neurodegeneration enlarged the local ECS width and increased nanoscale diffusivity in close vicinity of microglia and confirmed that HA is the main diffusion barrier in the brain ECS.

Our findings are in agreement with the study of Sherpa et al. (2020) who also found enlarged ECS and reduced water diffusion rates in the corpus callosum of conditional knock-out mice lacking HA synthase 2 (Has2). Using the RTI method in brain slices and DW-MRI in vivo, they found an increased volume fraction α and a decreased ADCw, which corresponded to electron-microscopical changes in white matter. In contrast to our in vivo study, they have not found changes in the brain gray matter and claim that HA has a different function in the gray and the white matter. Arranz et al. (2014) found a decrease of ECS volume in stratum pyramidale of the hippocampus and increased excitability of CA1 neurons in brain slices from Has3 knock-out mice, suggesting different effects of HA synthases subtypes. Increased TMA^+^ diffusion rates along and across the axons with no change in ECS volume were found in the corpus callosum of Bral1-deficient mouse (Cicanic et al., 2012). HAPLN4 deficiency in mice led to a decrease in ECS volume only in aged brains (Cicanic et al., 2018; Suchá et al., 2020). In vivo studies in Tenascin-R and in HNK-1-sulphotransferase knock-out mice revealed a decrease in ECS volume using both RTI and DW-MRI (Syková et al., 2005a). The disruption of ECM in knock-out animals reflects permanent changes during the whole lifespan and can result from developmental disorders manifested not only by dysregulation of ECM but also by changes in neuronal and astrocytic networks. Studies performed in knock-out animals are, therefore, different from our in vivo study where the 4-MU diet was applied in adult animals for a relatively short time period, with the aim to change ECM transiently. In vivo reductions in MD and FA and changes in MR spectroscopy suggest that astrocytes are altered after dysregulation of ECM. The observed changes in metabolites and significant changes in astrocyte-related parameters were detected at the same time points, at 4 and 6 months after 4-MU diet. The changes in GFAP staining and in myoinositol point to the role of astrocytes in ECM remodeling. Astrocytes facilitate the remodeling of ECM as they are the key regulators of ECM and PNN functions (Tewari et al., 2022). Astrocytes produce HA, various CSPGs, as well as matrix-remodeling proteases, such as HA cleaving enzyme hyaluronidase (Wiese et al., 2012), which suggests an important role of astrocytes in regulating the architecture and geometry of ECS. HA, in turn, regulates the morphology of astrocytes (Peters and Sherman, 2020), and, therefore, the inhibition of HA synthesis by 4-MU could induce changes in astrocytic network.

MR spectroscopy did not reveal major changes in the examined key brain metabolites, such as NAA (a marker of functional neurons), suggesting no substantial neuronal damage. The lower level of astrocyte marker mIns/tCr in the somatosensory cortex after 4 months of 4-MU diet may reflect the observed changes in astrocytic network. We can further speculate that reorganization/retraction of astrocytes and a decrease in PNNs may result in increased release of glutamate, as the Glu/tCr level has become significantly higher after 6 months of 4-MU diet. However, the sensitivity of fitting the macromolecular background to global parameters of the fitting algorithm urges some caution. While the performed measurements confirmed the absence of significant metabolic changes, the achieved signal-to-noise ratio may not sufficiently guarantee unbiased estimates of metabolites whose multiplets interfere with the macromolecular background (mIns/Tau, Glu/Gln).

It is important to note that diffusion in the ECS is largely anisotropic, i.e., different in different directions. The decrease of FA after 4-MU treatment as revealed by DW-MRI suggests changes in ECS microstructure and geometry. Such a decrease of FA might be related to a decrease of diffusion in certain preferential direction(s) due to HA fragmentation, loss of PNNs, and shortening and/or reorganization of astrocytic processes. Reduced astrocytic coverage of magnocellular neurons in the supraoptic nucleus of lactating rats led to a decrease in ECS volume and a loss of anisotropy, which facilitated diffusion and glutamate spillover, monitored by electrophysiological recordings of metabotropic glutamate receptor-mediated depression of GABAergic transmission (Piet et al., 2004). Loss of anisotropy, related loss of PNNs, and disorganization of astrocytic processes in the rat hippocampus were previously found during aging and were accompanied by a learning deficit (Syková et al., 2005b).

Astrocytes and PNNs regulate plasticity during development and in adulthood and stabilize synapses and inhibit plasticity in adult brain. Both transient and permanent depletion of PNNs in the somatosensory cortex of adult mice induced astrocytic structural plasticity around parvalbumin neurons without altering synapses, and PNN degradation impaired glutamate uptake and K^+^ buffering by astrocytes at tripartite synapse (Tewari et al., 2024). PNNs have been proposed to stabilize synapses and long-term memory by wrapping neuronal soma and proximal dendrites (Pizzorusso et al., 2002; Tsien, 2013; van 't Spijker and Kwok, 2017). Removal of PNNs disrupted the recall of a remote fear memory (Thompson et al., 2018), and cerebellar plasticity and associative memories were suggested to be controlled by PNNs (Carulli et al., 2020). PNN production is triggered by sensory stimulation and future experiences (Wang and Fawcett, 2012). Astrocytes and PNNs prevent spillover of GABA and glutamate and may, therefore, decrease formation of new synaptic contacts (Syková, 2004). Removal of PNNs that form a diffusion barrier between cells and ECS can facilitate spillover of transmitters from “naked” synapses to form new synaptic contacts (Syková, 2004). The PNN-free neurons and dendrites are more easily reached by molecules diffusing in the enlarged ECS (Fig. 8). PNNs have been also proposed to act as local barriers preventing neurons from an excess of cations in ECS (Hartig et al., 1999). Diffusion restriction by PNNs has been postulated in a recent study describing a reduced diffusion in a charged, course-grained planar polymer brush (spacings smaller than 10 nm), a model of diffusion in PNNs (Hanssen and Mathe-Sørenssen, 2022). However, the restriction of transport near neuronal surface is not yet understood. These findings together with our current diffusion studies and analysis of astrocyte morphology strongly point to the role of ECM remodeling in CNS plasticity.

Decreased ECS volume, deposits of substances and altered ECM during pathological states and aging can limit plasticity due to compromised extrasynaptic volume transmission, diffusion of neurotransmitters, neuroactive molecules, growth factors, drugs, and clearance of waste products from the brain (Bohr et al., 2022). The increased ECS volume and decreased FA after a 4-MU diet resemble the diffusion parameters found during early postnatal development (Lehmenkühler et al., 1993). Partial removal of PNNs by a 4-MU diet had some beneficial effect after spinal cord injury (Irvine et al., 2023; Stepánková et al., 2023). We suggest that the changes in ECS volume may support cell migration and axonal growth. Here we showed that a relatively short-term treatment of adult rats with HA synthase inhibitor 4-MU, which removed some but not all PNNs, led to a substantial increase in the ECS volume. Manipulation of PNNs and dECM and changes in ECS volume and geometry might be beneficial during treatment of brain diseases by opening plasticity, facilitating cell migration, growth of axons, and formation of new synaptic connections in adulthood.
